# Displacement Pattern of Anterior and Posterior Column Fragments in Both-Column Acetabular Fractures: A 3D Reconstruction-Based Study

**DOI:** 10.1155/2022/3556357

**Published:** 2022-10-20

**Authors:** Pengyu Ye, Junfei Guo, Zhongzheng Wang, Siyu Tian, Kuo Zhao, Yingchao Yin, Zhiyong Hou, Yingze Zhang

**Affiliations:** ^1^Department of Orthopaedic Surgery, Third Hospital of Hebei Medical University, Shijiazhuang, Hebei, China; ^2^Key Laboratory of Biomechanics of Hebei Province, Shijiazhuang, Hebei, China; ^3^NHC Key Laboratory of Intelligent Orthopaedic Equipment, Shijiazhuang, Hebei, China; ^4^Chinese Academy of Engineering, Beijing, China

## Abstract

**Background:**

Few studies have been conducted to examine the displacement characteristics of both-column acetabular fractures (BCAFs). The goal of this study was to investigate the displacement pattern of anterior column (AC) and posterior column (PC) fragments in BCAFs using 3D virtual software.

**Materials and Methods:**

BCAFs were retrospectively reviewed, and 81 patients were enrolled. Computed tomography (CT) images were imported into Mimics software. A 3D model of each pelvis was generated. Four marked points and the rotation angle of each of the injured ACs and PCs were identified. The fracture fragments were reduced virtually using the software, and the change in coordinates of the marked points after reduction was recorded while the rotation angle was measured. The measurements of positional and directional displacement were analysed using the Mann–Whitney *U* test and the binomial test, respectively.

**Results:**

There was a propensity for AC fragments to shift superomedially and to rotate externally (*p* < 0.001). Additionally, the posteroinferior fracture area of AC fragments showed the greatest displacement (*p* < 0.05). PC fragments moved superomedially (*p* < 0.001) and moved more at the proximal end than the distal end (*p* < 0.001). PC displacement was always accompanied by internal rotation (*p* < 0.001). Greater AC displacement was observed in the fracture area further away from the acetabulum (*p* < 0.05). Greater rotation was observed for the AC than the PC (*p* < 0.001).

**Conclusion:**

After a BCAF occurs, there are regular patterns regarding the direction and distance of AC and PC fragment displacement. Information on these patterns may provide insight into the injury mechanism and fracture morphology and facilitate surgical decision-making for orthopaedic trauma surgeons.

## 1. Introduction

Acetabular fractures, especially severe both-column fractures, are caused by high-energy injuries such as car accidents and falls from heights [1, 2]. Both-column acetabular fractures (BCAFs) account for approximately 20% of all types of acetabular fractures [1, 3]. After an injury occurs, two major fracture lines separate the acetabulum, and AC and PC fragments appear [4, 5], the spatial displacement of which is complicated. Anatomical reduction is the most vital component for a good outcome [6, 7]. However, even for senior orthopaedic trauma surgeons, it is still a challenge to achieve adequate reconstruction [8]. To obtain perfect fracture reduction, surgeons need to be familiar with the patterns of AC and PC fragment displacement.

Even though 3D imaging techniques are available, the displacement of fracture fragments is nevertheless regularly measured on X-rays or 2D computed tomography (CT) projections [9]. Due to the complex anatomy of the pelvis and inconsistencies in posture during the imaging examination, the reliability of the measurements is greatly reduced [10]. With the application of computerized 3D imaging software such as Mimics, fractures can be reconstructed virtually, and variations in pelvic posture can be fully taken into account, improving the accuracy of measurements [11].

In recent literature, virtual 3D software has been increasingly used to enable the evaluation of fracture morphology [12, 13]. However, unfortunately, there have been few reports on the displacement characteristics of BCAFs, much less the application of 3D software to examine them [14, 15]. In this study, we propose an innovative method by simulating the reduction of BCAFs in 3D software, measuring the change in both the coordinates of marked points after fracture reduction and the angle of fragment rotation to analyse the patterns of AC and PC fragment displacement in BCAFs.

## 2. Materials and Methods

### 2.1. Patients

A total of 81 eligible fractures were screened out by three independent investigators at a level I trauma emergency centre from April 2015 to October 2019. The collection criteria were as follows: (I) closed unilateral BCAF; (II) age>18 years; (III) no congenital disease related to the hip joint and no history of hip trauma or surgery. The exclusion criteria were as follows: CT slice thickness>2 mm and pathological fracture. Approval for this study was obtained from the institutional review board of this trauma centre (G2020-029-1).

### 2.2. Reduction and Measurements

Mimics 20.0 software (Materialise, Belgium) is an interactive medical imaging control software that can import data from CT and magnetic resonance imaging (MRI) scans and create 3D models for processing. Reduction and measurements were mainly conducted with the aid of Mimics and its affiliated 3-Matic software.

Each BCAF patient's pelvic CT Digital Imaging and Communications in Medicine (DICOM) data were imported into Mimics. The “Segment → CT Bone Segmentation” function was applied to generate a pelvic mask, which contained all fracture fragments. If the mask of an individual fragment was not separated, it could be separated with the assistance of the “Segment → Split Mask” function. Next, a 3D model of all fragments was reconstructed using the “Segment → Calculate 3D by Mask” function. All 3D models were then exported into 3-Matic.

**Figure 1:**
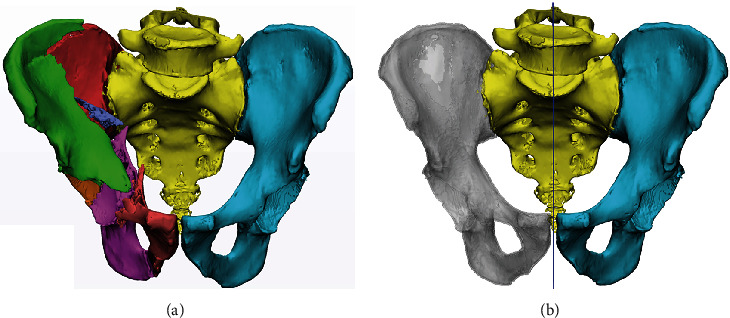
Standard pelvic model and reduction template. (a) Standard pelvic model accessible via “Interactive Rotate”. Different colours represent different individual units. (b) Reduction template (grey area, 25% transparency) created by “mirroring” the healthy hemipelvis (cyan area) with the median sagittal plane (blue line) as the axis of symmetry.

Due to the postural deviation of patients during the CT examination, each 3D model was adjusted to the standard position by the “Align → Interactive Translation and Rotate” function. The requirements for the standard position were as follows:

- *Axial Plane*. Parallel to the line connecting the right and left posterior superior iliac spines or sacral foramen with the same ordinal number
- *Sagittal Plane*. Perpendicular to the axial plane, passing through the median sacral crest and coinciding with the median sagittal plane
- *Coronal Plane*. Perpendicular to the sagittal and axial planes. When observed in the standard 3D image, the tip of the coccyx aimed at the pubic symphysis (Figure 1(a))

Before simulating the reduction of the AC and PC fragments, a reduction reference template had to be created. Previous studies have indicated a high degree of symmetry on both sides of the pelvis [16, 17]. Therefore, the healthy side of the pelvis was symmetrical to the injured side, and the fracture reduction template was obtained using the “Align → Mirror” function. The transparency of the template was changed to 25% using “Properties → Visualization → Transparency” (Figure 1(b)). Except for the AC and PC fragments, all parts were hidden.

**Figure 2:**
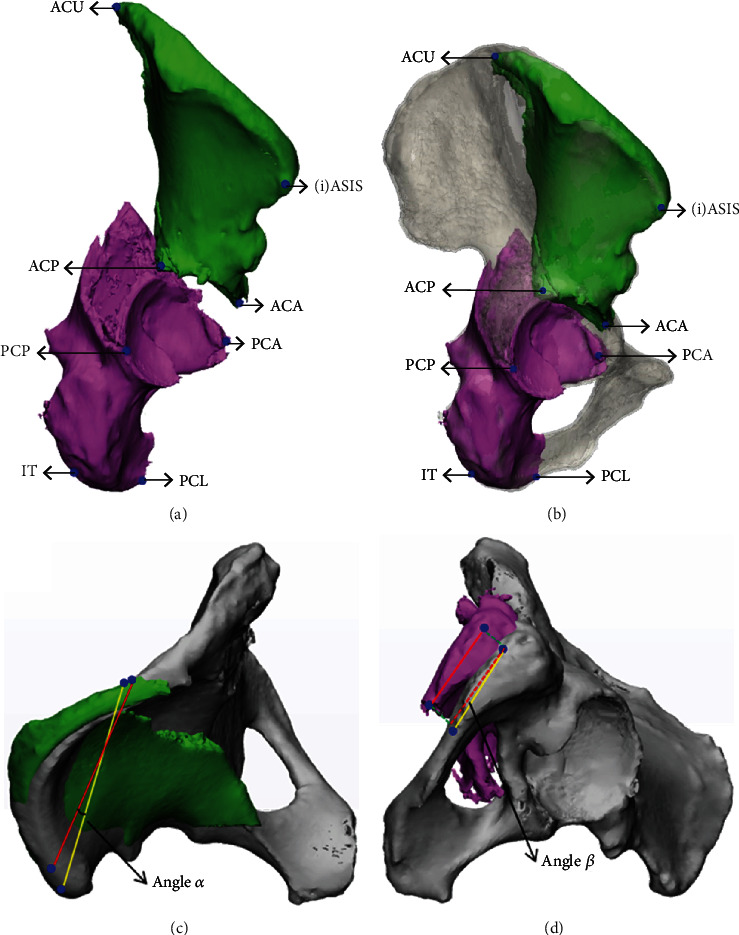
Marked points and rotation angles. (a) Nonreduced marked points on AC and PC fragments. (b) Reduced marked points on AC and PC fragments. (c) Top view showing angle *α* projected by lines between (i)ASIS and ACU before and after AC fragment reduction. (d) Bottom view showing angle *β* projected by lines between IT and PCL before and after PC fragment reduction (blue points, marked points; green area, AC fragment; purple area, PC fragment; grey area, reduction template; red line, connection between marked points before reduction; dashed red line, parallel to red line; yellow line, connection between marked points after reduction).

The AC fragment contained a posterior fracture line and an inferior fracture line, and the PC fragment contained an anterior fracture line and a superior fracture line. The lateral view was switched using the “View” function to create the four marked points, including three points on the fracture lines and an anatomical marked point on the AC or PC fragment, using “Analyze → Create Point”. On the AC fragment, the following steps were taken: (I) the point of the anterior superior iliac spine (ASIS), or the point inferior to the ASIS (iASIS) when the ASIS was not part of the AC fragment, was selected; (II) the uppermost point of the posterior fracture line was selected as the AC upper (ACU) point; (III) the lowermost point of the posterior fracture line was selected as the AC posterior (ACP) point; (IV) the most anterior point of the lower fracture line was selected as the AC anterior (ACA) point (Figure 2(a)). On the PC fragment, the following steps were taken: (I) the point of the ischial tuberosity (IT) was selected; (II) the uppermost point of the anterior fracture line was selected as the PC anterior (PCA) point; (III) the lowermost point of the anterior fracture line was selected as the PC lower (PCL) point; (IV) the point of the upper fracture line located most posterior was selected as the PC posterior (PCP) point (Figure 2(a)). The coordinates of these nonreduced points were displayed and recorded using the “Properties” function. Next, the reduction template was displayed. Based on the template, the AC and PC fragments with marked points were reduced using the “Align → Interactive Translate and Rotate” and “Registration” functions (Figure 2(b)), and then the coordinates of each point were recorded.

The differences in the coordinates of each marked point between before and after fracture reduction were calculated. The values of the calculated results represent the displacement distance for each marked point on the coronal plane (x-axis), sagittal plane (y-axis), and axial plane (z-axis), with positive and negative values indicating the direction of displacement, i.e., medial and lateral movement, posterior and anterior movement, and superior and inferior movement, respectively. The displacement distance in space was obtained according to the Euclidean distance formula.

In the top view, the acute angle formed by the line of the ASIS (or iASIS) point and ACU point before and after reduction, defined as the rotation angle *α* of the AC fragment, could be observed (Figure 2(c)). In the bottom view, the acute angle formed by the line of the IT point and PCL point before and after reduction, defined as the rotation angle *β* of the PC fragment, could be observed (Figure 2(d)). The values of *α* and *β* were measured on the axial plane, with a positive or negative sign indicating the direction of rotation.

### 2.3. Data Analysis

Categorical variables are summarized as frequencies and percentages, and continuous variables are summarized as medians and interquartile ranges (IQRs). Statistical analyses were performed using R software (RStudio, 4.0.3, USA). The displacement distance values were compared by the Mann–Whitney *U* test. Displacement directions were evaluated with the binomial test. The statistical significance level was set at *p* < 0.05. Histograms were used to describe differences in marked points of the AC fragment, of the PC fragment, and between the AC and PC fragments.

## 3. Results

### 3.1. Population Data

**Figure 3:**
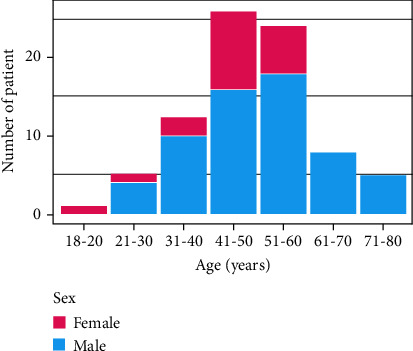
Distribution of fractures by patient age and sex.

A total of 81 eligible patients were enrolled. Patient demographic data are shown in Table 1. The peak incidence, which included 61.7% of the cases, was concentrated between the ages of 41 and 60 (Figure 3). Furthermore, the proportion of male patients (61, 75.3%) was higher than that of female patients (20, 24.7%).

### 3.2. AC Fracture Fragment

**Figure 4:**
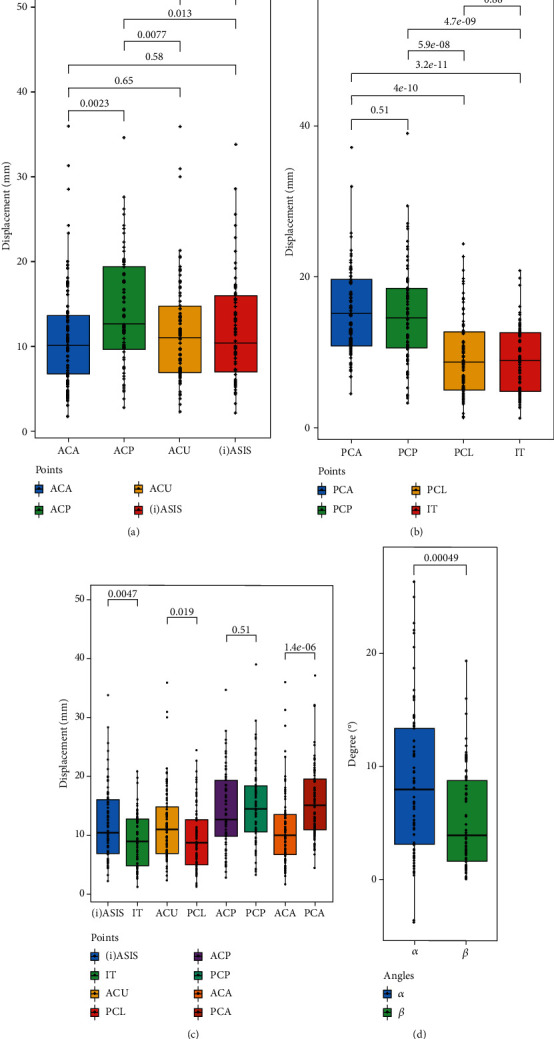
Comparison of marked points and rotation angles. (a) Comparison of ACA, ACP, ACU, and (i)ASIS for AC fragment. (b) Comparison of PCA, PCP, PCL, and IT for PC fragment. (c) Comparison of marked points of AC and PC fragments in terms of relative positions. (d) Comparison of rotation angles of AC and PC fragments.

In the AC fracture fragment, the fracture area near the impact centre of the acetabulum represented by the ACP and ACA points showed a characteristic displacement direction: superomedial displacement (ACP: x-axis 75, 92.6%, p < 0.001, binomial test; z-axis 77, 95.1%, p < 0.001, binomial test; ACA: x-axis 68, 84.0%, *p* < 0.001, binomial test; z-axis 75, 92.6%, *p* < 0.001, binomial test). However, at the top of the AC fragment, the direction of displacement was not as clear as at the impact centre. The (i)ASIS point showed an obvious tendency to move laterally (73, 90.1%, *p* < 0.001, binomial test), but the ACU point did not. The median rotation angle *α* was 7.93° (IQR: 10.40), and most of the rotation angle *α* signs were positive (78, 96.3%, *p* < 0.001, binomial test), which means that the AC fragments rotated externally (Table 2). Among the four marked points, the ACP point located at the posteroinferior articular zone of the AC fragment showed the most obvious shift [12.58 mm (IQR: 9.95), *p* < 0.05, Mann–Whitney *U* test]. In contrast, the shifts of the other three marked points were comparable (all *p* > 0.05, Mann–Whitney *U* test) (Figure 4(a)).

### 3.3. PC Fracture Fragment

In most cases, the four marked PC points moved superomedially, especially the PCA and PCP points (all *p* < 0.001, binomial test) (Table 3). The PCA and PCP points [PCA: 15.02 mm (IQR: 9.00), PCP: 14.51 mm (IQR: 7.97)] located at the top of the PC fragment showed greater displacement than the lower IT and PCL points [IT: 8.92 mm (IQR: 8.08), PCL: 8.67 mm (IQR: 7.88)] (all *p* < 0.001, Mann–Whitney *U* test) (Figure 4(b)). The median rotation angle *β* was 3.81° (IQR: 7.49), and all rotation angle *β* signs were positive (Table 3), demonstrating that PC fragments rotate internally.

### 3.4. AC vs. PC Fracture Fragments

According to the relative position of the four marked points on the AC and PC fragments, pairwise comparisons were made. The results showed greater displacement for the marked points in the extra-articular zone of the AC than the PC [(i)ASIS vs. IT: 10.39 mm (IQR: 9.28) vs. 8.92 mm (IQR: 8.08), *p* < 0.05, Mann–Whitney *U* test; ACU vs. PCL: 10.96 mm (IQR: 7.97) vs. 8.67 mm (IQR: 7.88), *p* < 0.05, Mann–Whitney *U* test]. However, in the injured anterior area of the acetabulum, the marked point of the PC shifted more (PCA vs. ACA: 15.02 mm (IQR: 9.00) vs. 10.10 mm (IQR: 7.06), *p* < 0.001, Mann–Whitney *U* test). There was no significant difference between the ACP and PCP (*p* = 0.51, Mann–Whitney *U* test) (Figure 4(c), Tables 2 and 3). Regarding rotation angles, the value of angle *α* was greater than that of angle *β* (7.93° (IQR: 10.40) vs. 3.81° (IQR: 7.49), *p* < 0.001, Mann–Whitney *U* test) (Figure 4(d), Tables 2 and 3), demonstrating greater rotation of the PC than the AC.

## 4. Discussion

In earlier studies, Letournel [1] and Brandser et al. [18] reported medial displacement of PC fracture fragments. Later, a study by Pierannunzii et al. [14] described that AC and PC fragments also showed external rotation and slight internal rotation, respectively. Nevertheless, they only presented these views and did not prove them. In this study, an innovative method was applied to explore the displacement of BCAF fragments. Several unique displacement patterns were revealed by quantitative and qualitative analyses. On the one hand, the AC and PC fragments showed similar displacement patterns, such as superomedial displacement of the fracture region located on the articular surface and rotational displacement of the fracture fragments; on the other hand, there were differences in the distance and direction of fracture fragment displacement and rotation.

**Figure 5:**
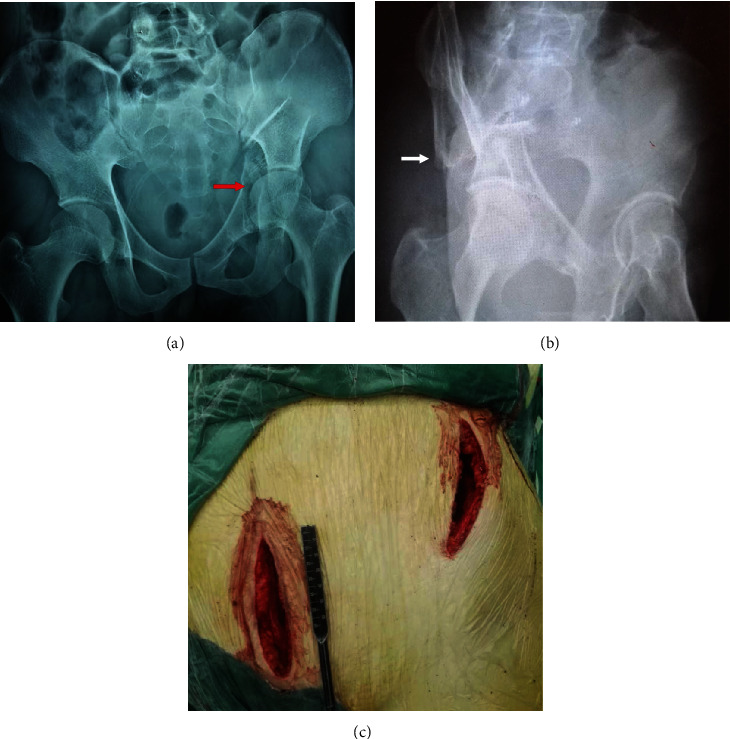
Characteristics of BCAFs. (a) Central dislocation of femoral head (red arrow). (b) “Spur sign” (white arrow). (c) Surgical incision for combined Stoppa+iliac fossa approach.

Approximately 90% of the marked points on the articular surface (ACA, ACP, PCA, PCP) showed superomedial displacement. The proportion of marked points showing medial displacement even reached 100% for the PC fragment. These fracture areas were close to the energy centre of the fracture and heavily displaced, indicating that the femoral head directly impacted the acetabulum in a medal and superior direction. Herman et al. reported a view similar to ours and classified BCAFs into the superomedial displacement vector group based on the superomedial injury mechanism [19]. Moreover, medial dislocation of the femoral head was not difficult to detect when the injury force was high (Figure 5(a)). The ACP point showed greater displacement than the other three marked points of the AC fragment and was consequently closer to the site of contact between the femoral head and acetabulum. In the proximal fracture area of the AC, the translation was undefined, and the displacement distance was minor relative to that in the injured articular zone. It may be possible that due to the long longitudinal fracture line of the AC, violence continuously evacuates during transmission from the ACP point up to the ACU point, offsetting the original displacement characteristics. Interestingly, 90.1% of the (i)ASIS points moved laterally, in the opposite direction of the medial injury force, which can be attributed to the passive influence of the external rotation of the AC fragment. As a parameter for the analysis of fracture fragment rotation, the rotation angle was introduced. In 96.3% of all cases, the AC fragment showed external rotation. Correction of the externally rotated AC is key to fracture repositioning; otherwise, it is impossible to restore the entire articular surface at the top of the acetabulum, thus impeding the subsequent reduction of the PC [20].

Overall, the direction of PC fragment displacement was fairly consistently superomedial. In addition to the impact force from the femoral head, the superomedial displacement is also exacerbated by the pulling of sacrotuberous and sacrospinous ligaments during PC detachment. Because of the proximity of the PCA and PCP points on the articular surface to the collision centre, these points showed a greater displacement distance than the PCL and IT points in the extra-articular zone. Many reports [1, 14, 21, 22] have mentioned that obturator oblique radiographs show the specific “spur sign” (Figure 5(b)) in cases of BCAFs; this sign is caused by the medial displacement of the PC fragment, while a piece of ilium remains in place, connected to the sacroiliac joint. Our results provide strong support for the principle of spur sign formation and additionally indicate that the AC and PC shift not only medially but also superiorly. In addition, all PC fragments showed internal rotation. The above displacement characteristics are all related to the injury mechanism: the anteromedial wall of the acetabulum is impacted by the femoral head during external rotation and abduction of the hip joint [14, 23].

We carried out a comparative study of the distance of AC and PC fragment displacement based on the location of the marked points and found less displacement of the extra-articular marked points of the PC. Additionally, the results showed less rotation of the PC than of the AC. However, the longitudinal fracture line of the PC was shorter than that of the AC, and the extra-articular marked points are closer to the acetabulum, which implies that the AC is subjected to a more superomedially oriented injury force. Another potential possibility is that the attachments of the powerful thigh adductor and posterior group muscles to the ischium and IT [24] confer some capability to resist displacement. In contrast, a comparison between the AC and PC on the anterior side of the acetabulum showed less displacement of the AC due to the attachment of the head of the rectus femoris, the iliocapsularis, and the iliofemoral ligament around the anterosuperior acetabulum as well as the greater thickness of the hip capsule in this area, all of which contribute to greater AC stability [25, 26]. Among all enrolled cases, the displacement of the marked points on the joint surface of the AC and PC fragments was more than 3 mm; however, a displacement distance of less than 3 mm is key to a good prognosis [27]. Therefore, a surgical approach for open reduction remains the best option for treating displaced acetabular fractures. Based on the displacement characteristics and our experience, the vast majority of procedures can be performed via a single anterior approach, such as the combined Stoppa and iliac fossa approach (Figure 5(c)) [28].

The widely used classification systems, including the Letournel-Judet and AO/OTA classification systems, only show the fracture location and do not specify more details regarding fracture displacement, particularly in multifragment cases [9]. However, the displacement characteristics clarified in this research can be used to supplement the above classification systems and provide a better understanding of BCAF morphology. To the best of our knowledge, this is the first study to demonstrate and characterize in greater detail both the direction and distance of AC and PC fragment displacement and rotation in BCAFs using 3D coordinate measures. Moreover, this method of investigating fracture displacement can be applied to other types of fractures. This study also has some limitations. The technique of using virtual 3D reconstruction software to simulate fracture reduction also has inherent limitations, as the reconstructed fracture fragments may not fit the template accurately. Thus, the measured results may deviate from the real values because the manual acquisition of marked points lacks sufficient accuracy [11]. Further biomechanical tests should be conducted in the future to verify the relationship between fracture displacement characteristics and injury mechanisms.

In conclusion, there are patterns of AC and PC fracture fragment displacement after a BCAF occurs: the AC fragment moves superomedially with external rotation, and the PC fragment moves superomedially with internal rotation. Additionally, the degree of rotation is greater for the AC than the PC. These displacement characteristics can assist orthopaedic surgeons in learning about fracture mechanisms and morphologies to plan better treatment strategies.

## Figures and Tables

**Table 1:** Patient demographic characteristics.

Demographic	Data
Mean age, year (range)	49.1 (20-74)
Sex, no. (%)	
Male	61 (75.3)
Female	20 (24.7)
Injured side, no. (%)	
Left	30 (37.0)
Right	51 (63.0)
Mechanism of injury, no. (%)	
High-energy trauma	79 (97.5)
Low-energy trauma	2 (2.5)

**Table 2:** Displacement distance and direction of marked points and rotation angle for the AC fragment.

Parameter	Distance^†^ (mm or °)	Direction^‡^		*p* value^∗^
		Positive sign	Negative sign	
(i)ASIS point				
x-axis	-5.74 (9.82)	8 (9.9)	73 (90.1)	<0.001
y-axis	4.03 (7.56)	61 (75.3)	20 (24.7)	<0.001
z-axis	-1.80 (8.85)	32 (39.5)	49 (60.5)	0.075
Spatial	10.39 (9.28)	—	—	
ACU point				
x-axis	-2.67 (9.95)	33 (40.7)	48 (59.3)	0.119
y-axis	-2.19 (9.60)	24 (29.6)	57 (70.4)	<0.001
z-axis	2.30 (6.01)	58 (71.6)	23 (28.4)	<0.001
Spatial	10.96 (7.97)	—	—	
ACP point				
x-axis	9.41 (10.16)	75 (92.6)	6 (7.4)	<0.001
y-axis	-0.82 (5.99)	34 (42.0)	47 (58.0)	0.182
z-axis	6.93 (7.48)	77 (95.1)	4 (4.9)	<0.001
Spatial	12.58 (9.95)	—	—	
ACA point				
x-axis	4.21 (5.61)	68 (84.0)	13 (16.0)	<0.001
y-axis	-4.08 (7.15)	18 (22.2)	63 (77.8)	<0.001
z-axis	5.54 (5.82)	75 (92.6)	6 (7.4)	<0.001
Spatial	10.10 (7.06)	—	—	
Angle *α*	7.93 (10.40)	78 (96.3)	3 (3.7)	<0.001

^†^, Data are given as the median and interquartile range (IQR). ^‡^, Data are presented as counts (percentages). ^∗^, Binomial test was applied for the displacement direction.

**Table 3:** Displacement distance and direction of marked points and rotation angle for the PC fragment.

Parameter	Distance^†^ (mm or °)	Direction^‡^		*p* value^∗^
		Positive sign	Negative sign	
IT point				
x-axis	4.60 (10.72)	62 (76.5)	19 (23.5)	<0.001
y-axis	-1.17 (6.11)	33 (40.7)	48 (59.3)	0.119
z-axis	2.88 (4.68)	63 (77.8)	18 (22.2)	<0.001
Spatial	8.92 (8.08)	—	—	
PCL point				
x-axis	6.04 (8.85)	71 (87.7)	10 (12.3)	<0.001
y-axis	-0.02 (6.33)	38 (46.9)	43 (53.1)	0.657
z-axis	1.81 (4.09)	62 (76.5)	19 (23.5)	<0.001
Spatial	8.67 (7.88)	—	—	
PCA point				
x-axis	12.52 (8.29)	81 (100)	0 (0)	<0.001
y-axis	1.20 (5.04)	49 (60.5)	32 (39.5)	0.075
z-axis	5.16 (5.47)	71 (87.7)	10 (12.3)	<0.001
Spatial	15.02 (9.00)	—	—	
PCP point				
x-axis	11.71 (8.35)	81 (100)	0 (0)	<0.001
y-axis	-0.34 (5.94)	38 (46.9)	43 (53.1)	0.657
z-axis	6.10 (7.01)	73 (90.1)	8 (9.9)	<0.001
Spatial	14.51 (7.97)	—	—	
Angle *β*	3.81 (7.49)	81 (100)	0 (0)	<0.001

^†^, Data are given as the median and interquartile range (IQR). ^‡^, Data are presented as counts (percentages). ^∗^, Binomial test was applied for the displacement direction.

## Data Availability

The data used to support the findings of this study are available from the corresponding author upon request.
